# LD Hub: a centralized database and web interface to perform LD score regression that maximizes the potential of summary level GWAS data for SNP heritability and genetic correlation analysis

**DOI:** 10.1093/bioinformatics/btw613

**Published:** 2016-09-22

**Authors:** Jie Zheng, A Mesut Erzurumluoglu, Benjamin L Elsworth, John P Kemp, Laurence Howe, Philip C Haycock, Gibran Hemani, Katherine Tansey, Charles Laurin, Early Genetics and Lifecourse Epidemiology (EAGLE) Eczema Consortium, Beate St Pourcain, Nicole M Warrington, Hilary K Finucane, Alkes L Price, Brendan K Bulik-Sullivan, Verneri Anttila, Lavinia Paternoster, Tom R Gaunt, David M Evans, Benjamin M Neale

**Affiliations:** 1 MRC Integrative Epidemiology Unit, University of Bristol, Oakfield House, Bristol, UK; 2 Genetic Epidemiology Group, Department of Health Sciences, University of Leicester, Leicester, UK; 3 University of Queensland Diamantina Institute, Translational Research Institute, Brisbane, QLD, Australia; 4 Department of Epidemiology, Harvard T.H. Chan School of Public Health, Boston, MA, USA; 5 Program in Medical and Population Genetics, Broad Institute of MIT and Harvard, Cambridge, MA, USA; 6 Analytical and Translational Genetics Unit, Department of Medicine, Massachusetts General Hospital and Harvard Medical School, Boston, MA, USA

## Abstract

**Motivation:**

LD score regression is a reliable and efficient method of using genome-wide association study (GWAS) summary-level results data to estimate the SNP heritability of complex traits and diseases, partition this heritability into functional categories, and estimate the genetic correlation between different phenotypes. Because the method relies on summary level results data, LD score regression is computationally tractable even for very large sample sizes. However, publicly available GWAS summary-level data are typically stored in different databases and have different formats, making it difficult to apply LD score regression to estimate genetic correlations across many different traits simultaneously.

**Results:**

In this manuscript, we describe LD Hub - a centralized database of summary-level GWAS results for 173 diseases/traits from different publicly available resources/consortia and a web interface that automates the LD score regression analysis pipeline. To demonstrate functionality and validate our software, we replicated previously reported LD score regression analyses of 49 traits/diseases using LD Hub; and estimated SNP heritability and the genetic correlation across the different phenotypes. We also present new results obtained by uploading a recent atopic dermatitis GWAS meta-analysis to examine the genetic correlation between the condition and other potentially related traits. In response to the growing availability of publicly accessible GWAS summary-level results data, our database and the accompanying web interface will ensure maximal uptake of the LD score regression methodology, provide a useful database for the public dissemination of GWAS results, and provide a method for easily screening hundreds of traits for overlapping genetic aetiologies.

**Availability and Implementation:**

The web interface and instructions for using LD Hub are available at http://ldsc.broadinstitute.org/

**Supplementary information:**

Supplementary data are available at *Bioinformatics* online.

## 1 Introduction

There is now substantial empirical evidence demonstrating that the majority of complex traits and diseases in humans are influenced by hundreds if not thousands of genetic loci of small effect scattered across the genome as was first predicted a century ago (East, 1916; Fisher, 1918). The advent of high throughput micro-array genotyping and now next generation sequencing technologies has meant that genome-wide data can be leveraged to ask fundamental questions concerning the underlying genetic architecture of common complex traits and diseases including the degree to which genetic variation affecting complex phenotypes is tagged by SNPs on genome-wide arrays (Lee et al., 2011; Yang *et al.*, 2010, 2011), the degree to which this variation represents different functional categories and/or biological pathways (Finucane *et al.*, 2015; Gusev *et al.*, 2014), and the extent to which genetic aetiologies are shared across different phenotypes (Bulik-Sullivan *et al.*, 2015b; Lee *et al.*, 2012). To date most of these types of analyses have been performed using genetic restricted maximum likelihood analysis (GREML) as implemented in software packages such as GCTA and LDAK (Lee et al., 2011; Speed et al., 2012; Yang *et al.*, 2010, 2011). However, these methods require individual-level genotype data, which is often not available as most of the largest GWAS analyses are conducted through meta-analyses, and so typically only report summary results statistics (Zheng *et al.*, 2013). Additionally GREML can be computationally prohibitive when analyzing raw genome-wide SNP data from hundreds of thousands of individuals. Consequently, most GREML analyses reported in the literature to date have been hypothesis driven studies that have involved only a small number of related traits (Table 1).

**Table 1.** Comparison between GREML and LD Score Regression via LD Hub

GREML	LD Score regression via LD Hub
Requires individual-level data	Requires GWAS summary-level data
One dataset at a time	Integrates multiple GWAS results datasets
Run time depends on number of individuals and traits	Run time depends on number of traits only
Manual implementation	Automated
Usually one or a few traits at a time	Many traits simultaneously
Typically hypothesis driven Computationally prohibitive for large numbers of individuals	Hypothesis driven or hypothesis-free Handles large numbers of individuals easily

In order to address these limitations, Bulik-Sullivan *et al* previously proposed a different method, LD score regression (Bulik-Sullivan *et al.*, 2015a). Essentially the method involves regressing summary results statistics from millions of genetic variants across the genome on a measure of each variant’s ability to tag other variants locally (i.e. its ‘LD score’). The intuition behind the approach is that if a trait is genetically influenced, then variants that tag more of the genome (i.e. have high LD scores) should have a greater opportunity to tag causal variants and therefore have higher test statistics on average than variants that have low LD scores. In this way genome-wide inflation of test statistics due to genuine polygenicity can be distinguished from biases such as population stratification and cryptic relatedness. The basic method is very flexible and can be adapted to estimate SNP heritability, calculate a more accurate and efficient genome-wide inflation correction factor than genomic control (Bulik-Sullivan *et al.*, 2015a), partition the SNP heritability by functional category (Finucane *et al.*, 2015), and estimate the genetic correlation between different complex traits and diseases (Bulik-Sullivan *et al.*, 2015b), all using GWAS summary-level results data (Table 1).

The chief limitation of using LD score regression to estimate genetic correlations to date has been a practical one. Publicly available GWAS meta-analysis results are available from a number of different repositories on the Internet. It is time consuming to locate and download all of these resources for use, particularly as these databases become more numerous. What’s more, each summary results file typically involves different file formats and conventions making data preparation a time consuming exercise. In addition, many GWAS meta-analyses are not made publicly available, requiring the user to proactively invite the relevant investigators to share their results, which also takes a significant amount of time.

Here we describe a centralized database and web interface, LD Hub, which automates the LD score regression analysis pipeline using publically available GWAS summary-level data of individuals with European ancestry. Users of our web-based tool only need to upload summary results for their trait(s) of interest; and the web server will automatically test their results against GWAS results from (currently) 173 other traits/diseases. The proposed database and web interface calculates the SNP heritability for the uploaded phenotype(s), and a genetic correlation matrix across traits. LD Hub allows the user to conduct the analysis on specific phenotypes only or perform a hypothesis free screen across all traits in the database (Table 1). Users have the option of uploading their own results files and the option of adding their GWAS results to the database for inclusion in future releases. The resource is continuously updated and curated every month to include new results from users and publicly available sources alike. The pre-computed genetic correlation matrix will be provided on LD-Hub for all traits included in the database.

## 2 Methods

**Fig. 1.**
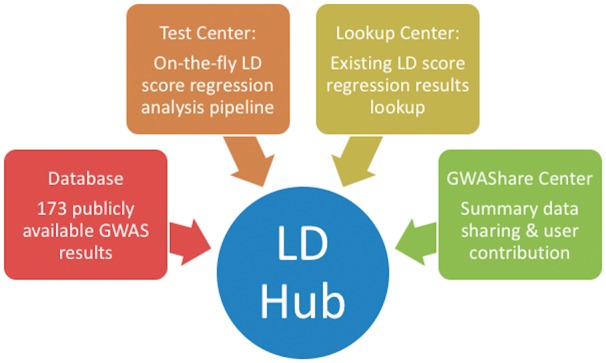
Scope and features of LD Hub. The LD Hub server provides three features: (i) Test Centre, which is an automatic LD score regression platform, (ii) Lookup Center, which allows users to lookup LD score regression results for their trait(s) of interest and (iii) GWAShare Center, which allows users to share their GWAS summary results and contribute to the field

As summarized in Figure 1, LD Hub includes: (1) Lookup Center: a facility to perform lookups of existing LD score regression results; (2) Database: a GWAS summary-level statistics database, (3) Test Center: a web interface that automates the LD score regression analysis pipeline including the calculation of SNP heritability and genetic correlations and (4) GWAShare Center: a user contribution and data sharing platform

### 2.1 LD Hub database

#### 2.1.1 GWAS summary-level data

We cleaned and harmonized 963 publicly available GWAS summary-level datasets from 36 consortia, which included 82 diseases, 154 complex traits, 576 metabolites and 151 immune markers (Hemani *et al*, in preparation).

From this database pool, we chose datasets that fit the following selection criteria:

1. Non-sex-stratified
2. Meta-analyses of predominantly European populations. We include a few GWAS meta-analyses that contain a small proportion of non-European individuals in them in the LD Hub database. Whilst we believe the effect of these small numbers of non-European individuals on the LD Score regression analyses will be relatively minor, users should be aware that results from these meta-analyses may be less robust because of inconsistent patterns of linkage disequilibrium between individuals of different ancestry. In order to flag these studies to the user, we have included an additional field in the Test Center and the GWAShare Center (last column) that indicates the population ancestry of individuals in the corresponding meta-analysis, as well as a similar field in the LD Score regression results file (see also Table S1).
3. Meta-analyses using a GWAS backbone chip only (i.e. exclude meta-analyses involving immuno | metabo | psych | exome chip or GWAS + custom chip)
4. Number of SNPs is large (N > 450 000)
5. Number of individuals is large (N > 5000)
6. Mean Chi-square of the test statistics is larger than 1

**Fig. 2.**
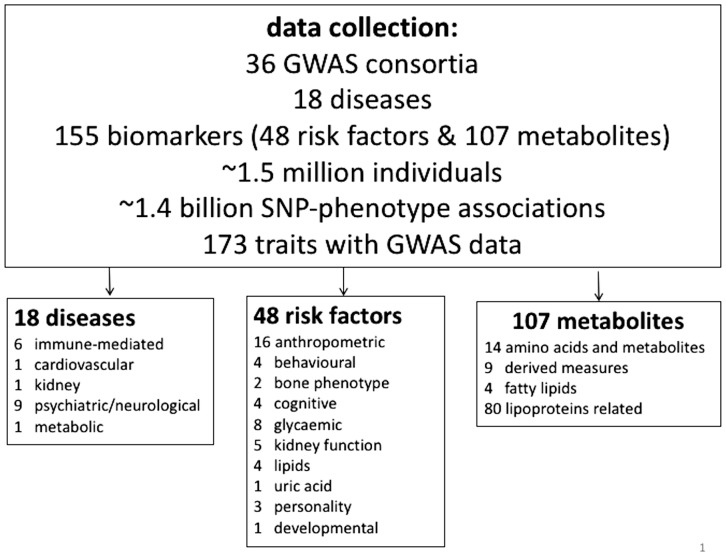
Contents of LD Hub. In total, data for 173 traits are included in LD Hub, which consist of 18 diseases, 48 complex traits and 107 metabolites

As shown in Figure 2, after filtering on the selection criteria, genome-wide results for 173 traits were included in LD Hub, of which 18 are GWAS of diseases (Boraska *et al*., 2014; Cross-Disorder Group of the Psychiatric Genomics Consortium, 2013; Lambert *et al*., 2013; Liu *et al* 2015; Moffatt *et al*., 2007; Morris *et al*., 2012; Neale *et al*., 2010; Nikpay *et al*., 2015; Okada *et al*., 2013; Paternoster *et al*., 2015; Ripke *et al*., 2012; Ripke *et al*., 2014; Simon-Sanchez *et al*., 2009; Sklar *et al*., 2011), 48 are medically relevant risk factors/complex traits (Benyamin *et al*., 2013; Berndt *et al*., 2013; Bradfield *et al*., 2012; Dastani *et al*., 2012; de Moor *et al*., 2010; Dupuis *et al*., 2010; Estrada *et al*., 2012; Furberg *et al*., 2010; Horikoshi *et al*., 2012; Huffman *et al*., 2015; Lango Allen *et al*., 2010; Manning *et al*. 2012; Moffatt *et al*., 2007; Pattaro *et al*., 2016; Perry *et al*., 2014; Rietveld *et al*., 2014; Rietveld *et al*., 2013; Saxena *et al*., 2010; Shungin *et al*., 2015; Soranzo *et al*., 2010; Speliotes *et al*., 2010; Taal *et al*., 2012; Teslovich *et al*., 2010; Teumer *et al*., 2016; van den Berg *et al*., 2014; van der Valk *et al*., 2014) and 107 are metabolites (Kettunen *et al*., 2016). Table S1, displays descriptive information for each of the GWAS in LD Hub, including, trait name, consortium name, ethnicity, gender, sample size, PubMed ID, year of publication and other relevant information.

#### 2.1.2 LD score information

We pre-calculated LD scores for each SNP using individuals of European ancestry from the 1000 Genomes project (1000 Genomes Project Consortium, 2012). These LD scores are suitable for standard LD score analyses in European populations (i.e. the LD score regression intercept, heritability, genetic correlation, cross-sex genetic correlation).

### 2.2 LD Hub web interface

The LD Hub web interface framework was developed using Python Django v1.8 as the LD score regression program is written using Python.

#### 2.2.1 Test center

**Fig. 3.**
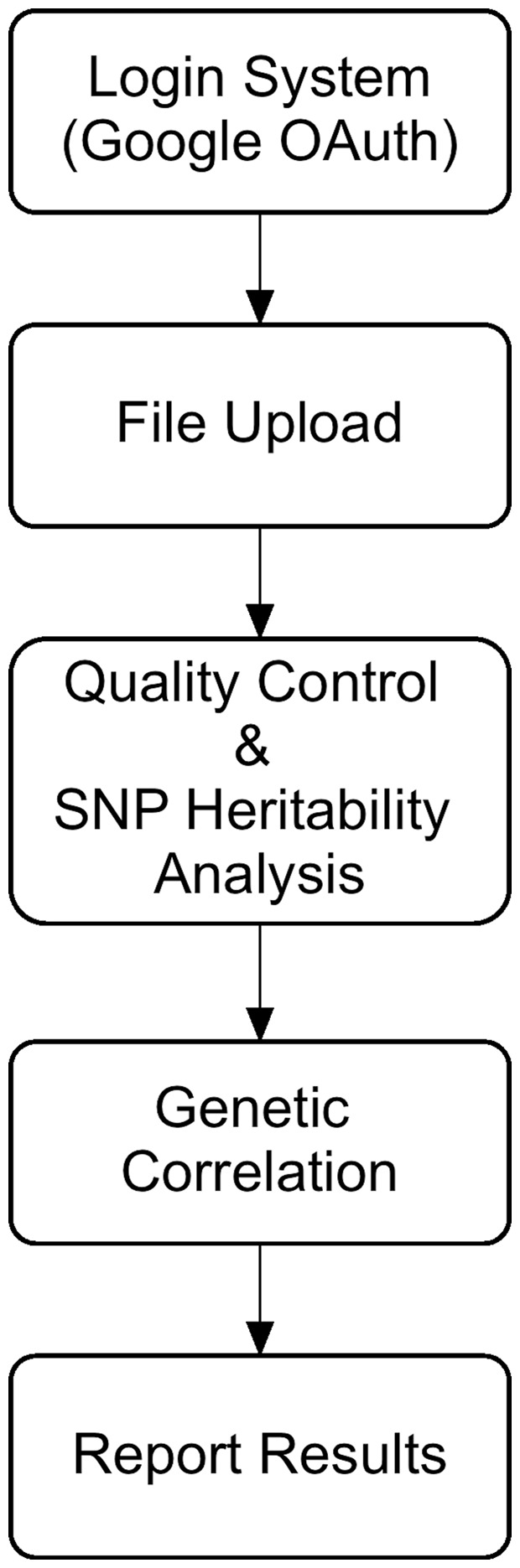
Schematic of LD Hub workflow. To start using LD Hub, users are required to login using a GMail (compatible) account. Once logged in, the users can then navigate their way around, selecting the features and databases they are interested in

The LD Hub web interface provides an automatic LD score regression analysis pipeline for users. As shown in Figure 3, the LD Hub analysis pipeline consists of 5 major steps:

1. User login system: using a Google OAuth system (login by using a Google account)
2. File upload system: To run the LD score analysis pipeline, LD Hub requires upload of a file containing summary results data. In the web interface, we provide an example GWAS results file to illustrate the file format required for successful upload and analysis by LD Hub. To save uploading time, each results file should be a white space delimited zipped text file (LD Hub accepts both tab and/or space delimited zipped text files) in which each row contains the results from a single SNP whilst the columns comprise the following fields:
  - SNP ID (rs number)
  - Effect allele of the SNP
  - Alternate allele of the SNP
  - Sample size of each SNP (can use an overall sample size if sample size for some SNPs is missing)
  - A signed summary statistic where the sign refers to the addition of the effect allele (i.e. any statistic that can be converted into a *Z*-score)
  - P value of the SNP
  - Minor allele frequency of each SNP (optional)
  - SNP Imputation quality (optional)
3. Quality control and heritability analysis: To standardize the input file, quality control is automatically performed on the uploaded file.
  - For studies that provide sample MAF, a filter to include SNPs with MAF above 1%.
  - In order to restrict the analysis to well-imputed SNPs, we filter the uploaded SNPs to HapMap3 SNPs (International HapMap 3 Consortium *et al.*, 2010) with 1000 Genomes EUR MAF above 5%, which tend to be well-imputed in most studies. In the future, as the ability to impute lower frequency SNPs improves we will investigate the possibility of including other SNPs in the analysis using resources like the Haplotype Resource Consortium (HRC).
  - If sample size varies from SNP to SNP, remove SNPs with an effective sample size less than 0.67 times the 90th percentile of sample size.
  - Remove insertions and deletions (INDELs) and structural variants.
  - Remove strand-ambiguous SNPs.
  - Remove SNPs whose alleles do not match those in the 1000 Genomes data.
  - Remove SNPs within the major histocompatibility complex (MHC) region (i.e. SNPs between 26Mb and 34Mb on chromosome six) since these often display extreme LD and/or effect sizes. Inclusion of these outlying SNPs would have the potential to bias results of SNP heritability and genetic correlation analyses similar to the inclusion of outliers in traditional regression analyses and would therefore be inappropriate.
  - Because outliers can unduly influence the regression, we also removed SNPs with extremely large effect sizes (*X*_1_^2^ > 80). The second part of this step is the SNP heritability analysis. The results of this analysis provide a useful indication of whether genetic correlation analysis is likely to be informative (Bulik-Sullivan *et al.*, 2015b). We recommend that users restrict subsequent genetic correlation analyses to GWAS that achieve a *Z* score of at least four in SNP heritability analyses on the grounds of interpretability and power. Genetic correlations that are derived from GWAS with *Z* scores < 4 are flagged with a note (Table S1).
4. Genetic correlation analysis. The LD Hub pipeline will perform genetic correlation analysis on the uploaded GWAS results after the SNP heritability analysis. Users have the option of selecting which traits they want to include in the analysis. Occasionally LD Hub will produce estimates of the genetic correlation that exceed positive or minus one. Often these estimates will involve GWAS that are small in size, exhibit low SNP heritability *Z* scores (we recommend *Z* scores > 4 to be interpretable), and large standard errors around the genetic correlation estimate. We advise the user to treat these estimates as unreliable and discard them. In contrast, it is also possible for genetic correlation estimates to exceed one if the analysis involves two very similar traits from large GWAS that exhibit good power (e.g. GWAS of body mass index and obesity). In this case, the true genetic correlation is probably one and the user is advised to interpret the results accordingly.
5. Reporting of results. LD Hub returns three output (results) files to the users: (i) A log file with quality control information with regards to the uploaded GWAS summary data; (ii) A ‘h2.log’ file with the SNP heritability information about the uploaded GWAS data and (iii) A ‘rg.results.csv’ file with pair-wise genetic correlations between the uploaded GWAS results and the selected GWASs in LD Hub.

#### 2.2.2 Lookup center

Another feature of the LD Hub web interface is the heritability and genetic correlation ‘lookup’ function for GWAS results which currently exist in the LD Hub database. In the current version (v1.0), we provide (i) SNP heritability and (ii) genetic correlation results. Both, tables and downloadable links can be found on the Lookup Center webpage.

#### 2.2.3 GWAShare center

We aim to promote sharing of summary GWAS results data and to this extent we have included web links to all the publicly available summary GWAS results data that we have incorporated into LD Hub (users will find this link in the GWAShare Center along with a PubMed identifier detailing which study the data came from). In the case of summary results GWAS data that are not publicly available outside LD Hub, users will need to get in touch with the authors of the study themselves to request the data. Users may find this feature useful in conducting other types of SNP comparative study outside the scope of LD Hub as well as following up interesting genetic correlations. We encourage users of LD Hub to upload their GWAS results for curation into the database. We will update the database regularly and allow other users to use the shared data for LD score regression analyses, which will then benefit the whole human genetics community.

### 2.3 LD Hub applied example: atopic dermatitis

In order to illustrate the utility of LD Hub, we conduct an analysis using summary results data from a large GWAS of atopic dermatitis (AD) for 40 835 (10 788 cases and 30 047 controls, sample prevalence: 0.264) individuals of European ancestry (i.e. the whole discovery set except 23andMe results) (Paternoster *et al.*, 2015). In total, 11 059 640 SNPs were included in this meta-analysis. Since AD is influenced by a gene of major effect, i.e. filaggrin—variants in this region have allelic odds ratios > 7 (Sandilands *et al.*, 2007), which could bias estimates from LD Hub, we excluded this region from the uploaded results file. For traits/diseases that have a single locus of disproportionately large effect (i.e. χ^2 ^>^ ^80) compared to the rest of the genome, we recommend the exclusion of SNPs in these regions as good practice when using LD Hub (and LD score regression in general), since the inclusion of these SNPs could unduly leverage the regressions and consequently the estimates of genetic correlations and SNP heritability. However, with the exception of autoimmune diseases (SNPs in the MHC can have large effects on certain autoimmune diseases), it is unusual for common traits/diseases to exhibit a single locus of large effect, and thus this potential source of bias should not be an issue for a majority of diseases/traits. For traits that exhibit a single locus of disproportionately large effect (χ^2 ^>^ ^80), we recommend fine-mapping and direct evaluation of overlap in the particular region to assess whether genetic effects are shared, and LD score regression of the rest of the genome with this particular region excluded from analyses. After the abovementioned quality control steps, 1 215 002 SNPs were selected for upload.

## 3 Results

### 3.1 Validation of LD Hub analysis results

We tested the validity and functionality of LD Hub by replicating previously reported results from the original LD Score regression suite of papers (Bulik-Sullivan *et al.*, 2015a, b).

We compared SNP heritability results between LD Hub and previously reported LD score regression results (Bulik-Sullivan *et al.*, 2015a). As shown in Table S2, the Mean *χ*^2^, *λ*_GC_ and Intercept results are almost the same. The minor discrepancies observed are a consequence of using slightly different quality control processes for LD Hub compared to what was used in the original LD Score regression paper. Results for the SNP heritability of 173 traits are shown in Table S1.

**Fig 4:**
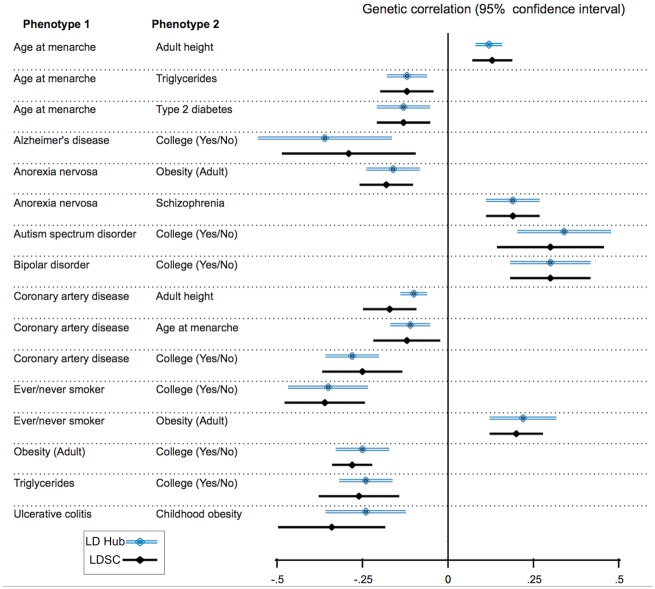
Comparison of genetic correlation results between LD Hub and previously reported LD score regression results. Double blue lines represent genetic correlation results from LD Hub, and the black single lines represent genetic correlation results from previously reported LD score regression results. The discrepancies can be attributed to the minor changes in the quality control processes and the replacement of some GWAS results with more recent versions

We also compared the genetic correlation analysis results between LD Hub and previously reported results (Bulik-Sullivan *et al.*, 2015b). As shown in Figure 4, the genetic correlation and standard error of genetic correlation estimates are consistent with previously reported LD score regression genetic correlation results. A comparison of the genetic correlation results of 49 (previously reported) traits is shown in Table S3.

### 3.2 Case study: atopic dermatitis

Table 2 shows SNP heritability estimates for AD computed with and without SNPs from the filaggrin region. The figure of 7.8% (9.7%) is low particularly compared to the heritability estimates from twin studies of eczema where figures exceeding 80% are not uncommon (Bataille *et al.*, 2012). This could be for a number of reasons including the fact that genomic control correction in the individual meta-analysis studies causes downward bias, and the fact that LD score regression provides an estimate of the overall proportion of additive genetic variance tagged by SNPs in the GWAS panel (i.e. SNP heritability), rather than total heritability *per se*. However the greatest contributing factor is likely to be the case definition of AD used in the EAGLE consortium paper (http://www.wikigenes.org/e/art/e/348.html) which is extremely heterogeneous, relying often on self-reported data or retrospective recall which will introduce substantial measurement error into the analysis (and hence decrease heritability estimates). Our results strongly suggest that reanalysis using a more precise definition of eczema would result in a cleaner phenotype and consequently increase the number of genome-wide significant loci detected.

**Table 2.** SNP heritability for atopic dermatitis

Type of Heritability Scale	H^2^	SE_H^2^	*λ*_GC_	Mean *χ*^2^	Intercept
Observed Scale (no filaggrin)	0.071	0.016	1.053	1.080	1.034
Liability Scale (no filaggrin)	0.078	0.018	1.053	1.080	1.034
Observed Scale (with filaggrin)	0.073	0.018	1.054	1.083	1.034
Liability Scale (with filaggrin)	0.097	0.020	1.054	1.083	1.034

H^2^ and SE_H^2^ refer to the SNP heritability and standard error of the SNP heritability.

Table 3 displays estimated genetic correlations between AD and several immune mediated diseases recorded in LD Hub. As expected, the estimated genetic correlation (rG) between AD and asthma was strongly significant and positive. We also note that the rG between AD and Crohn’s disease was moderate, significant and positive, perhaps reflecting substantial overlap between currently known loci for both conditions (Paternoster *et al.*, 2015). rG did not differ significantly from zero for the other traits, although the point estimates for several were moderate indicating that follow up when larger samples become available may be justified.

**Table 3:** Genetic correlation between atopic dermatitis and other immune mediated diseases

Traits	rG	SE_rG	P_rG
Crohn's disease	0.18	0.09	0.03
Ulcerative colitis	0.10	0.10	0.31
Asthma	0.55	0.15	0.0002
Rheumatoid Arthritis	−0.07	0.08	0.40

rG refers to the genetic correlation between two traits, SE_rG is the standard error of the genetic correlation, P_rG is the p value of the genetic correlation.

## 4 Discussion

In this paper, we describe LD Hub (accessible at http://ldsc.broadinstitute.org/), a web-based utility that centralizes and harmonizes summary-level GWAS results data, and automates LD Score regression analysis (Bulik-Sullivan *et al.*, 2015a, b).

GWAS meta-analysis summary statistics are increasingly being made publicly available. Our database (currently) utilizes results from 173 different GWAS, which includes the majority of publicly available GWAS summary results suitable for LD Score regression (Bulik Sullivan *et al.*, 2015a). However, this represents a small proportion of the traits represented in the GWAS Catalog (https://www.ebi.ac.uk/gwas/) (Hindorff *et al.*, 2009; Welter *et al.*, 2014). There is thus an urgent need for increased sharing of GWAS meta-analysis results in order to realize the full potential of techniques that utilize summary results data such as LD score regression. LD Hub provides a natural platform for the distribution of summary results data that can be utilized by the whole genetics community.

There are four major advantages of using our database and web interface:

1. Users of LD Score regression currently spend most of their time reformatting, harmonizing and managing summary results data rather than running the ‘actual’ analyses. LD Hub minimizes the proportion of time spent on the former so that users can focus their attention on interpreting interesting genetic correlations and SNP heritability estimates.
2. Users who do not have a computational background will find the interface easier to use
3. The software is computationally very fast. The current version (v1.0) can return the systematic analysis results to the user within a few hours. A queuing system has been introduced to prevent the server from crashing.
4. As users upload and share their own summary GWAS results, the resource becomes increasingly useful.

We envisage LD Hub as a useful hypothesis generating tool, providing an easy method of screening hundreds/thousands of traits for interesting genetic correlations that could subsequently be followed up in further detail by other approaches such as pathway analysis (Segrè *et al.*, 2010) or Mendelian randomization (Davey-Smith and Ebrahim, 2003). For example, under most models, a causal relationship between two heritable traits should induce a genetic correlation between the two phenotypes (assuming individual differences in the causal trait are influenced by genetic variation). LD Hub could be used to screen a large number of putatively causally related phenotypes quickly and easily for evidence of genetic correlation, and the most promising candidate pairs could then be followed up by selecting appropriate genetic instruments and performing formal instrumental variables analysis (Evans Davey-Smith, 2015) which can be implemented via the online platform MR-Base (www.mrbase.org/beta). This framework could be particularly useful in the dissection of high dimensional molecular networks where the number of possible pair-wise relationships may be extremely large.

For LD Hub, we list a few suggestions/limitations here:

1. In order for estimates of the genetic correlation to be reliable we suggest that traits uploaded meet the following criteria
  - Heritability (H^2^) *Z* score is at least > 1.5 (optimal > 4)
  - Mean Chi square of the test statistics > 1.02
  - The intercept estimated from the SNP heritability analysis is between 0.9 and 1.1
2. As we aim to provide an analysis pipeline that is as systematic as possible, we used a very inclusive strategy for data selection, where we expect a small proportion of the analyses (especially for the traits with notes in Table S1) to return null results.
3. LD Hub is currently designed for GWAS studies involving European populations exclusively. As the number of publicly available GWAS involving other ethnicities increases we will extend LD Hub to include these.

In summary, due to the growing availability of summary-level data, our database together with the web interface will maximize the potential of GWAS summary-level data for heritability and genetic correlation analyses.

## Funding

This work was supported by the Medical Research Council (MC_UU_12013/4 and MC_UU_12013/8). This work was also supported by the following grants: 1R01MH101244-02 (Statistical methods for studies of rare variants) and 1R01MH107649-01 (Methods for linking GWAS peaks to function in psychiatric disease) D.M.E. is supported by an Australian Research Council Future Fellowship (FT130101709). This work was in part supported by Cancer Research UK programme grant number C18281/A19169 (the Integrative Cancer Epidemiology Programme). P.H. is a Cancer Research UK Population Research Fellow (grant number C52724/A20138).

*Conflict of Interest*: none declared.

## Supplementary Material

Supplementary Data
