# Supplementary material for: LD Hub: a centralized database and web interface to perform LD score regression that maximizes the potential of summary level GWAS data for SNP heritability and genetic correlation analysis
Source: Bioinformatics. 2016 Sep 22;33(2):272–9. doi: 10.1093/bioinformatics/btw613 (PMC5542030; doi:10.1093/bioinformatics/btw613)
Supplement: Supplementary Data [file btw613_supp.zip › btw613-suppl_data/Supplmentary_text.docx]

**Text S1. References for GWAS data included in the LD Hub v1.0**

Anderson,C. et al. (2011) Meta-analysis identifies 29 additional ulcerative colitis risk loci, increasing the number of confirmed associations to 47. Nature Genetics, 43, 246-252.

Bentham,J. et al. (2015) Genetic association analyses implicate aberrant regulation of innate and adaptive immunity genes in the pathogenesis of systemic lupus erythematosus. Nature Genetics, 47, 1457-1464.

Benyamin,B. et al. (2013) Childhood intelligence is heritable, highly polygenic and associated with FNBP1L. Molecular Psychiatry, 19, 253-258.

Berndt,S. et al. (2013) Genome-wide meta-analysis identifies 11 new loci for anthropometric traits and provides insights into genetic architecture. Nature Genetics, 45, 501-512.

Boger,C. et al. (2011) CUBN Is a Gene Locus for Albuminuria. Journal of the American Society of Nephrology, 22, 555-570.

Boraska,V. et al. (2014) A genome-wide association study of anorexia nervosa. Molecular Psychiatry, 19, 1085-1094.

Bradfield,J. et al. (2012) A genome-wide association meta-analysis identifies new childhood obesity loci. Nature Genetics, 44, 526-531.

Cordell HJ, et al. (2015). International genome-wide meta-analysis identifies new primary biliary cirrhosis risk loci and targetable pathogenic pathways. Nat Commun. 6:8019.

Dastani Z et al. (2012). Novel loci for adiponectin levels and their influence on type 2 diabetes and metabolic traits: a multi-ethnic meta-analysis of 45,891 individuals. PLoS Genet. 8(3):e1002607.

de Moor,M. et al. (2010) Meta-analysis of genome-wide association studies for personality. Molecular Psychiatry, 17, 337-349.

Dubois,P. et al. (2010) Multiple common variants for celiac disease influencing immune gene expression. Nature Genetics, 42, 295-302.

Dupuis,J. et al. (2010) New genetic loci implicated in fasting glucose homeostasis and their impact on type 2 diabetes risk. Nature Genetics, 42, 105-116.

Estrada,K. et al. (2012) Genome-wide meta-analysis identifies 56 bone mineral density loci and reveals 14 loci associated with risk of fracture. Nature Genetics, 44, 491-501.

Franke,A. et al. (2010) Genome-wide meta-analysis increases to 71 the number of confirmed Crohn's disease susceptibility loci. Nature Genetics, 42, 1118-1125.

Furberg,H. et al. (2010) Genome-wide meta-analyses identify multiple loci associated with smoking behavior. Nature Genetics, 42, 441-447.

Horikoshi,M. et al. (2012) New loci associated with birth weight identify genetic links between intrauterine growth and adult height and metabolism. Nature Genetics, 45, 76-82.

Huffman,J. et al. (2015) Modulation of Genetic Associations with Serum Urate Levels by Body-Mass-Index in Humans. PLOS ONE, 10, e0119752.

Identification of risk loci with shared effects on five major psychiatric disorders: a genome-wide analysis (2013) The Lancet, 381, 1371-1379.

KÃttgen,A. et al. (2010) New loci associated with kidney function and chronic kidney disease. Nature Genetics, 42, 376-384.

Lambert,J. et al. (2013) Meta-analysis of 74,046 individuals identifies 11 new susceptibility loci for Alzheimer's disease. Nature Genetics, 45, 1452-1458.

Lango Allen,H. et al. (2010) Hundreds of variants clustered in genomic loci and biological pathways affect human height. Nature, 467, 832-838.

Liu JZ, et al. (2015). Association analyses identify 38 susceptibility loci for inflammatory bowel disease and highlight shared genetic risk across populations. Nat Genet. 47(9):979-86.

Mahajan,A. et al. (2014) Genome-wide trans-ancestry meta-analysis provides insight into the genetic architecture of type 2 diabetes susceptibility. Nature Genetics, 46, 234-244.

Manning,A. et al. (2012) A genome-wide approach accounting for body mass index identifies genetic variants influencing fasting glycemic traits and insulin resistance. Nature Genetics, 44, 659-669.

Morris,A. et al. (2012) Large-scale association analysis provides insights into the genetic architecture and pathophysiology of type 2 diabetes. Nature Genetics, 44, 981-990.

Moffatt MF, et al. (2007). Genetic variants regulating ORMDL3 expression contribute to the risk of childhood asthma. Nature. 448(7152):470-3.

Neale,B. et al. (2010) Meta-Analysis of Genome-Wide Association Studies of Attention-Deficit/Hyperactivity Disorder. Journal of the American Academy of Child & Adolescent Psychiatry, 49, 884-897.

Okada,Y. et al. (2013) Genetics of rheumatoid arthritis contributes to biology and drug discovery. Nature, 506, 376-381.

Perry,J. et al. (2014) Parent-of-origin-specific allelic associations among 106 genomic loci for age at menarche. Nature, 514, 92-97.

Prokopenko,I. et al. (2014) A Central Role for GRB10 in Regulation of Islet Function in Man. PLoS Genetics, 10, e1004235.

Rietveld,C. et al. (2014) Common genetic variants associated with cognitive performance identified using the proxy-phenotype method. Proceedings of the National Academy of Sciences, 111, 13790-13794.

Rietveld,C. et al. (2013) GWAS of 126,559 Individuals Identifies Genetic Variants Associated with Educational Attainment. Science, 340, 1467-1471.

Ripke,S. et al. (2012) A mega-analysis of genome-wide association studies for major depressive disorder. Molecular Psychiatry, 18, 497-511.

Ripke,S. et al. (2014) Biological insights from 108 schizophrenia-associated genetic loci. Nature, 511, 421-427.

Sawcer,S. et al. (2011) Genetic risk and a primary role for cell-mediated immune mechanisms in multiple sclerosis. Nature, 476, 214-219.

Saxena,R. et al. (2010) Genetic variation in GIPR influences the glucose and insulin responses to an oral glucose challenge. Nature Genetics, 42, 142-148.

Schunkert,H. et al. (2011) Large-scale association analysis identifies 13 new susceptibility loci for coronary artery disease. Nature Genetics, 43, 333-338.

Shin,S. et al. (2014) An atlas of genetic influences on human blood metabolites. Nature Genetics, 46, 543-550.

Shungin,D. et al. (2015) New genetic loci link adipose and insulin biology to body fat distribution. Nature, 518, 187-196.

SimÃ³n-SÃ¡nchez,J. et al. (2009) Genome-wide association study reveals genetic risk underlying Parkinson's disease. Nature Genetics, 41, 1308-1312.

Sklar,P. et al. (2011) Large-scale genome-wide association analysis of bipolar disorder identifies a new susceptibility locus near ODZ4. Nature Genetics, 43, 977-983.

Soranzo,N. et al. (2010) Common Variants at 10 Genomic Loci Influence Hemoglobin A1C Levels via Glycemic and Nonglycemic Pathways. Diabetes, 59, 3229-3239.

Speliotes,E. et al. (2010) Association analyses of 249,796 individuals reveal 18 new loci associated with body mass index. Nature Genetics, 42, 937-948.

Stahl,E. et al. (2010) Genome-wide association study meta-analysis identifies seven new rheumatoid arthritis risk loci. Nature Genetics, 42, 508-514.

Taal,H. et al. (2012) Common variants at 12q15 and 12q24 are associated with infant head circumference. Nature Genetics, 44, 532-538.

Teslovich,T. et al. (2010) Biological, clinical and population relevance of 95 loci for blood lipids. Nature, 466, 707-713.

van den Berg,S. et al. (2014) Harmonization of Neuroticism and Extraversion phenotypes across inventories and cohorts in the Genetics of Personality Consortium: an application of Item Response Theory. Behav Genet, 44, 295-313.

van der Valk,R. et al. (2014) A novel common variant in DCST2 is associated with length in early life and height in adulthood. Human Molecular Genetics, 24, 1155-1168.

Yang,J. et al. (2012) FTO genotype is associated with phenotypic variability of body mass index. Nature, 490, 267-272.
